# Aortic Dissection and Renal Failure in a Patient with Severe Hypothyroidism

**DOI:** 10.1155/2012/842562

**Published:** 2012-07-09

**Authors:** Valerie Brooke, Sangeeta Goswami, Arpan Mohanty, Pashtoon Murtaza Kasi

**Affiliations:** ^1^Department of Physical Medicine and Rehabilitation, University of Pittsburgh Medical Center (UPMC), Pittsburgh, PA 15213, USA; ^2^International Scholars Program, Department of Internal Medicine, University of Pittsburgh Medical Center (UPMC), Pittsburgh, PA 15213, USA

## Abstract

Acute aortic dissection (AAD) is a life-threatening condition associated with high morbidity and mortality. The most important recognized acquired cause that leads to dissection is chronic arterial hypertension. With respect to the anuria and renal failure, aortic dissection is not something that is always considered and is still not a very common presentation unless both renal arteries come off the false lumen of the dissection. However, when present, preoperative renal failure in patients with acute type B dissection has been noted to be an independent predictor of mortality. Early recognition and diagnosis is the key and as noted by previous studies as well, almost a third of these patients are initially worked up for other causes until later when they are diagnosed with aortic dissection. Here we present a case of a patient presenting with severe hypothyroidism, long-standing hypertension, and anuria. Through the case, we highlight the importance of having aortic dissection as an important differential in patients presenting with anuria who have a long standing history of uncontrolled hypertension. Pathophysiology relating to severe hypothyroidism-induced renal dysfunction is also discussed.

## 1. Case Presentation

The patient is a 68-year-old male with history of untreated hypothyroidism, untreated hypertension, and no medical care for over the last 10 years who presented to hospital with complaints of nausea, vomiting, and lower extremity weakness. Patient had called 911 two weeks prior for an episode of chest pain that felt like he was having a heart attack. When emergency medical service (EMS) arrived, chest pain had resolved and patient refused to come to hospital. A similar episode of severe chest pain occurred the following week, for which he called 911, but again refused transfer. On the day of admission patient called 911 again, but this time for nausea, vomiting, and weakness. When EMS arrived, they noticed he had slurred speech, a left-sided facial droop, and, therefore, transferred him to the hospital with concerns for stroke.

In the emergency room, physical exam was most remarkable for all the classic signs of hypothyroidism including hypothermia at 35.8°C, periorbital edema, puffy facies, macroglossia, hoarse voice, and delayed relaxation of deep tendon reflexes. His electrocardiogram (EKG) showed low voltage and sinus bradycardia with a rate in the 40 s. He did have left-sided facial droop and dysarthria, which was found to have been present for many years according to his family, and strength was 5/5 throughout his upper and lower extremities. No other focal neurological deficits were appreciated. Head CT without contrast indicated there was no acute intracranial pathology, brain MRI without contrast showed extensive chronic microvascular ischemic disease, as well as remote microhemorrhages in the right occipital and left cerebellar hemisphere. Lumbar spine MRI without contrast showed multilevel degenerative changes, most pronounced at the L5-S1 with a diffuse disc bulge, moderate-to-severe left and right neural foraminal stenosis, but no central canal stenosis.

Initial laboratory data was significant for a TSH of 63.4 IU/mL, creatinine of 1.9 mg/dL, hemoglobin of 7.3 gm/dL, and a normal white blood cell count. Patient was given two units of packed red blood cells, which improved his anemia to 9.7 gm/dL. He was admitted to general medicine service for further management of his severe hypothyroidism and workup for his anemia of unknown etiology.

The following morning repeat labs showed further decline in his kidney function, with a creatinine of 3.1 mg/dL, and potassium of 5.1 mMol/L. There also was new leukocytosis of 15 (×10^9^/L) with a 94% left shift, a new thrombocytopenia of 131 (×10^9^/L), down from 225 (×10^9^/L) at admission, and an elevated creatine phosphokinase (CPK) of 500 IU/L. A portable chest X-ray did not show any obvious sings of widened mediastinum but did show a left lower lobe consolidation consistent with a pneumonia for which he was started on IV azithromycin and ampicillin/sulbactam.

Nursing staff noted stool incontinence, for which a rectal exam was performed showing good rectal tone, and a positive guaiac. In addition, despite receiving aggressive fluid resuscitation, patient continued to be in auric renal failure. Patient then received 3 more liters of fluid throughout the day, a Foley was placed, and bladder scans showed a total of 48 cc of urine, enough to send urine studies. Urinalysis was negative for any signs of infection, and urine electrolytes indicated a fractional excretion of sodium (FeNa) of 0.96% looking initially like a prerenal process.

Labs were again repeated that evening, with a rising creatinine to 4.1 mg/dL, a lactate of 3.7 mMol/L, and patient still had no urinary output. Nephrology and endocrinology specialists were consulted, and the thought process was that his renal failure was likely stemming from his severe hypothyroidism causing a low flow state. He was started on levothyroxine (T4) and liothyronine (T3) and continued to get intravenous fluids.

The third day after admission morning laboratory data showed further increase in his creatinine to 6.1 mg/dL, a worsening leukocytosis to 16.7 (×10^9^/L), an improved lactate of 2.2 mMol/L, and a worsening thrombocytopenia of 92 (×10^9^/L). Thrombotic thrombocytopenia purpura (TTP) and HUS were also considered on the differential, given the anemia, and high LDH of 1014 IU/L. However, the smear did not have significant amounts of schistocytes, and the haptoglobin was normal; thus making it less likely.

**Figure 1:**
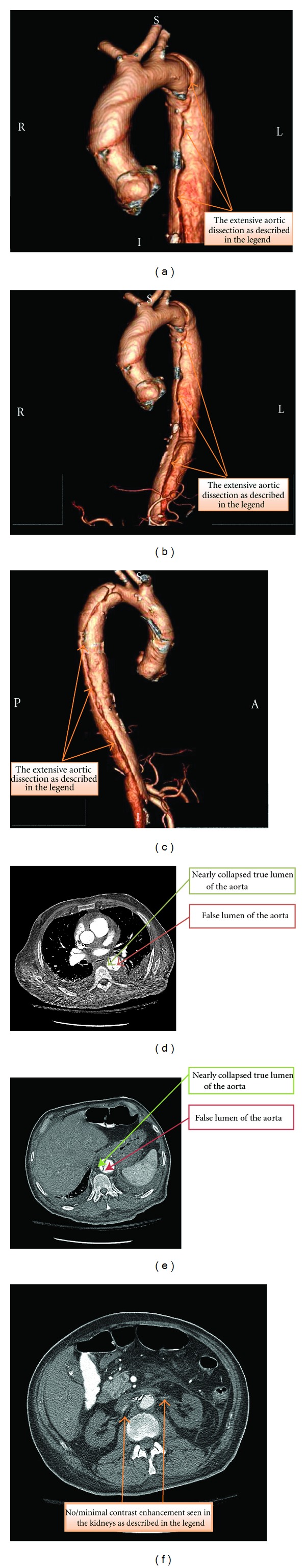
(a)–(c) Show the 3-dimensional reconstructs of the aortic dissection, with (d), (e), and (f) showing cross-sectional images through different parts showing the dissection. In summary, the CT angiogram demonstrated that there was a Type B Aortic dissection beginning in the descending thoracic aorta in the distal arch just past the origin of the subclavian artery. The true lumen was small in size and anterior right aspect of the aorta with a larger false lumen. The dissection extends into the abdominal aorta as shown in (a)–(c). As mentioned, the dissection extends into the abdomen. The true lumen is nearly completely collapsed at the level of the celiac and superior mesenteric arteries. Minimal contrast enhancement is seen entering the right renal artery and no enhancement of the left renal artery is noted (f). Again, the distal aortic limb is nearly completely occluded with extension of the dissection in both common iliac arteries.

Patient began complaining of abdominal pain and in the setting of an increasing leukocytosis and diarrhea, an abdominal CT without contrast was performed. This showed colitis, which looked either infectious or ischemic, as well as, a possible aortic dissection. A CT angiogram of the chest, abdomen, and pelvis was subsequently performed STAT, which showed a large type B dissection starting in the descending thoracic aorta just past the origin of the subclavian artery, extending into the abdominal aorta, with near complete collapse of the true lumen at the level of the renal arteries, with extension of the dissection into the common iliac arteries bilaterally, and ending at the level of iliac bifurcation (see Figures 1(a)–1(f)).

Following is a discussion on the presentation of this case of severe hypothyroidism and long-standing hypertension who presented with anuria/renal failure. Through the case, we highlight the importance of having aortic dissection as an important differential in patients presenting with anuria who have a long-standing history of uncontrolled hypertension.

## 2. Discussion

“Acute aortic dissection (AAD) is a life-threatening condition associated with high morbidity and mortality [1]. It is not uncommon to come across patients with aortic dissection. While hourly mortality data for type B AAD are not available, the overall in-hospital mortality is reported to be 11%. For those patients in the highest risk group, type B mortality can be as high as 71% [1].” 

Aortic dissection is a variant of so-called Acute Aortic Syndromes (AASs), which include other variants including intramural hematoma (IMH) and atherosclerotic ulcer. With improved diagnostic modalities, these syndromes are diagnosed early and more often [2]. With respect to “intramural hematomas of the aorta, they usually result from rupture of the vaso vasorum within the medial wall, resulting in aortic infarction; and in a third or more of cases, IMHs evolve into aortic dissections. Most cases of IMH occur within the descending thoracic aorta in patients with chronic systemic arterial hypertension. Like AAD, IMH may extend up or down the aorta, regress (10% of cases), and reabsorb [2].” And as noted in our case, it is possible that he might have developed an IMH first that progressed to such an extensive dissection. His two episodes of chest pain prior to presenting to the hospital may have been indicative of this.

Descriptions of aortic pathologies including dissection date back to the 2nd century, but it was not until 1955 that the first successful management of a case of aortic dissection was reported by Ramanath et al. [2]. 

The most important recognized acquired cause that leads to dissection is chronic arterial hypertension, and as in our case, the uncontrolled hypertension explains the extensive dissection and intramural hematoma noted. Other associated causes include iatrogenic (cardiac procedures, intra aortic balloon pumps, etc.), pregnancy (third trimester and early post partum), and familial syndromes/connective tissue disorders (Table 1). There have also been case reports of patients with myxedema who had dissections, but we only came across 4 cases and whether it was a coincidence or a causal risk factor is questionable [3–5]. 

Patient with type A dissections usually present with chest pain, and type B dissections more so with back or abdominal pain. Abdominal pain is seen approximately a one-fifth of type A and about half of patients with type B dissections. As noted in our case as well, it was the worsening of the abdominal pain and nonresolving diarrhea that made us pursue abdominal imaging that showed the findings as noted.

Pulse deficits in one or more arterial vessels have important prognostic implication and in our patient as well, when a temporary dialysis line was being placed, the right femoral pulse specifically was really difficult to appreciate.

Most of the cases of aortic dissection described in the literature have been often referred to as missed and not being timely diagnosed. In a retrospective review of 49 patients in Greece, almost a third of patients were initially admitted for other reasons [11]. However, as noted by Ramanath et al. in a very comprehensive review on acute aortic syndromes, “a key point for clinicians is that nearly 30% of patients later found to have AAD are initially diagnosed as having other conditions”; as was the case in our patient as well; the severe hypothyroidism leading to possibly a low flow state and renal failure was the initial diagnosis and the thought process that initially did not lead us to think about aortic dissection. There was a delay of 72 hours before the aortic dissection was diagnosed.

Severe hypothyroidism can present with impairment of renal function. Pathophysiology underlying hypothyroidism-induced renal impairment is not completely understood. It is postulated that renal blood is compromised in hypothyroidism secondary to a hypodynamic state resulting in low glomerular filtration rate (GFR) and reduced tubular secretory and reabsorptive capacity [12]. Furthermore, glomerular morphological changes are also seen in profound hypothyroidism [13]. Moreover, rhabdomyolysis secondary to severe hypothyroid myopathy also contributes to impairment of renal function. In fact, acute renal failure seen in hypothyroidism is often attributed to the associated rhabdomyolysis [14–16]. In our patient as well, acute renal failure in the setting of severe hypothyroidism with increased CPK led us to consider it as a case of hypothyroidism induced renal failure. However, in retrospect CPK level in our patient was not as high as seen in typical cases of rhabdomyolysis. In addition to this, complete anuria has not been reported in hypothyroidism.

With respect to the anuria and renal failure, aortic dissection is not something that is always considered and is still not a very common presentation unless both renal arteries come off the false lumen of the dissection, as noted in our case [17]. Renal failure in this case was due to pure vascular compromise and not hypotension. Aldridge and Birchall reviewed the literature regarding dissecting aneurysm presenting as renal failure and did not find a lot of cases pertaining to it. Per the literature review by Aldrige, Demos et al. described three cases and found only four others in the literature [18–20]. Similarly, some degree of renal dysfunction was noted in up to 8% of patients in a single-center study of 272 patients, but again frank renal failure with anuria is not that commonly seen [21]. Woywodt and colleagues also reported a case of anuria in an otherwise healthy patient with hypertension, in whom too the diagnosis of AAD was diagnosed later in admission [22]. There was also a reported case of a patient who had a high-speed motor vehicle crash resulting in a traumatic mid-thoracic aortic dissection. Since it resulted in involvement of the orifices of both renal arteries, anuria was noted in that case [23]. In another retrospective study of all the cases who presented to a center at Greece, renal failure/anuria was not the main presenting feature [11]. However, when present, preoperative renal failure in patients with acute type B dissection was noted to be an independent predictor of mortality [24, 25]. 

There are several different imaging modalities that can be utilized in the diagnosis of an aortic dissection with varying degrees of sensitivities and specificities. Chest X-ray has a sensitivity of 67% and specificity of 70% for mainly thoracic aortic dissection, and the findings of a “widened mediastinum” are often missed, making it the least useful imaging modality [26]. Aortography, where contrast is injected into the femorals, used to be the study of choice for evaluating suspected aortic dissections many years ago, with a sensitivity ranging between 81–91% [27]. Computerized Tomography (CT) scan with contrast have varying degrees of sensitivity and specificity depending on the type of dissection. CT for acute Type A dissections is 80% and 94% sensitive for subacute and acute dissections, respectively, while CT for Type B dissections is 93% and 100% sensitive for subacute and acute dissections, respectively; whereas MRI has a reported sensitivity and specificity of 98% and 85%, respectively [28]. Transesophageal echo has also been studied and has a reported sensitivity between 97–99%, but with a lower specificity of 77–85% due to false positive findings in the ascending aorta [29]. More often than not, it is usually with CT scans with contrast/CT angiograms that a diagnosis is made or incidentally noted as in our case.

With respect to management in patients presenting with type B dissection, medical therapy usually is the primary mode of treatment in controlling their hypertension, and in cases where an intervention would be required, like in ours, endovascular has better outcomes and less morbidity/mortality associated with it [30, 31]. For acute Type B aortic dissections with renal, mesenteric, limb ischemia, or neurological deficits, open surgical or endovascular repair with stenting has a Class IIa recommendation, where there is conflicting evidence but in a favorable direction regarding the efficacy of intervention [32]. Open surgical repair consists of a left posterolateral thoracotomy with a prosthetic graft replacement of the descending thoracic aorta, with a reported 10–17% mortality rate [32]. Endovascular repair consists of an aortic endograft or stent placement that obliterates the false lumen, with significantly lower rates of in-hospital mortality as compared to open surgery, 11% versus 33% noted in one series [32]. In addition, the more complicated the dissection, the higher rate of mortality, with a 50–88% mortality rate for patients with an unstable Type B aortic dissection with renal or mesenteric ischemia. These lower morbidity and mortality figures with the endovascular repair persisted despite the procedure done in older patients in some series [33]. 

Renal failure, cerebrovascular accidents, paraplegia (temporary or permanent), access site injuries, and endovascular leaks are the usual categories of complications seen with these procedures and are noted to be reversible as well in most of these patients [34, 35]. These complications are noted to decrease in centers doing a higher volume of these cases, and outcomes improve as the learning curve gets better. Perioperative mortality estimates from these procedures are also variable (10–22% on some series) and have been relatively improving as noted above [36, 37]. 

Early diagnosis and intervention along with associated comorbities (with Acute Physiology and Chronic Health Evaluation (APACHE) II score as a general indicator of patient condition used in some series) appear to be the major contributing factors with the classic teaching of mortality figures increasing by 1% per hour in these patients as the diagnosis gets delayed [37, 38]. 

The other interesting thing from the management standpoint are reports of cases where renal function has been noted to recover up to even 2 months after the dissection leading to frank renal failure from compromised blood flow to the renal arteries [39]. 

With respect to our patient, initially an exploratory laparoscopy was pursued by the general and vascular surgeons to make sure that there was no bowel ischemia/gangrene given evidence of colitis and fluid in his pelvis with tenderness on exam concerning for possible perforation. These were not noted on the exploratory laparoscopy and bowels looked viable. An endovascular repair was then pursued. Patient initially was in the intensive care unit and was transitioned slowly to rehab given deconditioning from the prolonged hospital course. He continues to have dialysis (now through a permanent dialysis catheter) and thus far, 2 weeks after his initial hospitalization, his renal function has not returned.

This case highlights the importance of having aortic dissection as an important differential in patients presenting with anuria who have a long standing history of uncontrolled hypertension.

## 3. Conclusions

- A key point for clinicians is that nearly 30% of patients later found to have acute aortic dissections are initially diagnosed as having other conditions.
- With respect to the anuria and renal failure, aortic dissection is not something that is always considered and is still not a very common presentation unless both renal arteries come off the false lumen of the dissection.
- When present, preoperative renal failure in patients with acute type B dissection is noted to be an independent predictor of mortality.
- In patients presenting with type B dissection, medical therapy usually is the primary mode of treatment in controlling their hypertension and in cases where an intervention would be required, endovascular has better outcomes and less morbidity/mortality associated with it.

## Figures and Tables

**Table 1:** Predisposing factors and associations reported in patients with aortic dissection [6–10].

Hypertension^∗∗^
Atherosclerosis^∗∗^
Connective tissue Disorders:
Ehlers-Danlos syndrome
Marfan syndrome
Loeys-Dietz syndrome
Annuloaortic ectasia
Inflammatory diseases/Vasculitides:
Giant cell arteritis
Takayasu arteritis
Rheumatoid arthritis
Syphilitic aortitis
Procedural associations/Trauma:
After a coronary artery bypass graft surgery (CABG)
Previous aortic valve replacement
Cardiac catheterization
Trauma
Other associations:
Peripartum
Preexisting aortic aneurysm
Bicuspid aortic valve
Aortic coarctation
Turner syndrome
Crack cocaine

^∗∗^ Systemic hypertension is the most important predisposing factor, followed by atherosclerosis.
